# Precise Controlled Target Molecule Release through Light-Triggered Charge Reversal Bridged Polysilsesquioxane Nanoparticles

**DOI:** 10.3390/polym13152392

**Published:** 2021-07-21

**Authors:** Xin Zhang, Mengmeng Zhang, Mingyue Wu, Linchuan Yang, Rui Liu, Rui Zhang, Tongtong Zhao, Ci Song, Gang Liu, Qingzeng Zhu

**Affiliations:** Key Laboratory of Special Functional Aggregated Materials, Ministry of Education, School of Chemistry and Chemical Engineering, Shandong University, Jinan 250100, China; zhangxin201511553@163.com (X.Z.); 201812260@mail.sdu.edu.cn (M.Z.); mywu@mail.sdu.edu.cn (M.W.); 201932207@mail.sdu.edu.cn (L.Y.); 202032381@mail.sdu.edu.cn (R.L.); 202012323@mail.sdu.edu.cn (R.Z.); 202032410@mail.sdu.edu.cn (T.Z.); 201812257@mail.sdu.edu.cn (C.S.); sdu_lg@sdu.edu.cn (G.L.)

**Keywords:** polysilsesquioxane, photocleavage, molecules release, light-triggered, charge reversal, precise control

## Abstract

Precise control of target molecule release time, site, and dosage remains a challenge in controlled release systems. We employed a photoresponsive molecule release system via light-triggered charge reversal nanoparticles to achieve a triggered, stepwise, and precise controlled release platform. This release system was based on photocleavage-bridged polysilsesquioxane nanoparticles which acted as nanocarriers of doxorubicin loaded on the surface via electrostatic interaction. The nanoparticles could reverse into positive charges triggered by 254 nm light irradiation due to the photocleavage of the *o*-nitrobenzyl bridged segment. The charge reversal property of the nanoparticles could release loaded molecules. Doxorubicin was selected as a positively charged model molecule. The as-prepared nanoparticles with an average size of 124 nm had an acceptable doxorubicin loading content up to 12.8%. The surface charge of the nanoparticles could rapidly reverse from negative (−28.20 mV) to positive (+18.9 mV) upon light irradiation for only 10 min. In vitro release experiments showed a cumulative release up to 96% with continuously enhancing irradiation intensity. By regulating irradiation parameters, precisely controlled drug release was carried out. The typical “stepped” profile could be accurately controlled in an on/off irradiation mode. This approach provides an ideal light-triggered molecule release system for location, timing, and dosage. This updated controlled release system, triggered by near-infrared or infrared light, will have greater potential applications in biomedical technology.

## 1. Introduction

A controlled molecule release system holds the ability to trigger molecules release and maintain an effective concentration of molecules at the target site. However, precise control over the timing, location, and quantity of molecules released is still a challenge. Stimuli-responsive drug nanocarriers are supposed to overcome the challenge in drug release systems [1,2,3,4,5,6,7]. Light, with remote and on-demand control over irradiation parameters, has become a promising stimulus for drug release systems [8,9]. Therefore, a light-controlled drug release system is considered as an ideal platform for precise control of drug release behavior [10,11,12,13].

A direct method for precisely controlling the dosage of drugs is the covalent combination of photocleavable nanocarriers and target molecules [14]. The target molecules are covalently attached to nanocarriers containing photocleavable groups, such as *o*-nitrobenzyl and coumarinyl derivatives [15]. The photocleavage of nanocarriers by light leads to the removal of the drug molecules, giving precise control over the drugs. Yibing Zhao reported that carboxytetramethylrhodamine, as a drug model, could be covalently caged by *o*-nitrobenzyl linkage-modified silica nanoparticles through a direct esterification process and released precisely by light [16]. Linyong Zhu groups provided a mesoporous silica nanoparticle grafted by the phototrigger coumarin modified with the drug chlorambucil, which could precisely regulate drug release upon light manipulation [17]. Magnetic silica nanoparticles fabricated using a coumarin-chlorambucil covalent conjugate were used for the precise control of the photolytic release of chlorambucil [18]. Pradeep Singh grouped developed photoresponsive mesoporous silica nanoparticles for the precise controlled release of chlorambucil attached to a quinoline phototrigger covalently [19]. Whereas the covalent method still has nonnegligible defects that the complicated chemical preparation and multistep operation might affect the effective efficiency of molecules, so it is urgent to find a new strategy to overcome the drawback.

Other methods have been tried to construct light-controlled drug release systems, like photocleavable polymeric vesicles, micelles, or liposomes [20], the drugs are encapsulated into cores of these nanocarriers via hydrophilic and hydrophobic interactions. Upon irradiation, the encapsulated drugs were continuously released from the disrupted nanocarriers due to photolysis, which caused the poor controllability of drug release systems. Therefore, it is still necessary to develop a stable and efficient methodology for precisely controlled drug release.

Electrostatic interaction is an advanced technology to load and release drugs due to its generalizability and fast responsivity. At present, stimuli-responsive charge-reversal nanocarriers exhibit significant potential for controlled drug release systems [21,22,23,24]. For example, the surface charge of nanocarriers plays an important role in prolonged circulation and cellular uptake in the physiological environment. The negatively charged nanocarriers maintain their characteristics during circulation in the blood and then reverse their surface charge to enhance cellular uptake once stimulated [25]. Although charge-reversal nanocarriers triggered by endogenous stimuli (e.g., pH, redox, enzyme) have been constructed [26,27,28,29,30,31,32,33], the low precision of the release dosage is a problem that has to be faced in drug release systems. The concentrations of these stimuli in the physiological environment could not be optionally regulated and controlled. Meanwhile, light-responsive charge-reversal nanocarriers hold tremendous potential to be explored for precise control of drug release. If light-responsive and electrostatic interactions are combined, precisely controlled drug release can be achieved. It should be mentioned that light-responsive charge-reversal nanocarriers have been employed for delivering nucleic acid molecules [34,35,36,37]. However, the positive charge of pristine nanocarriers is not conducive to their circulation in the blood, resulting in the inapplicability of the drug release systems.

Bridged polysilsesquioxane has a general chemical structure of [RSiO_1.5_]_n_, in which R is an organic bridged group and [SiO_1.5_] is the inorganic framework of the polymers [38]. The organic bridged group (R) can be designed to endow the special properties of bridged polysilsesquioxane, including its stimuli-responsive properties. The inorganic framework of [SiO_1.5_] affords this polysilsesquioxane good thermal stability, solvent resistance and biocompatibility. Therefore, bridged polysilsesquioxanes can serve as excellent carriers [39] and are potential candidates for widespread applications, including delivery systems [40,41,42], photoelectric sensors [43,44], molecular recognition [45,46], and adsorbents [47].

In the present work, bridged polysilsesquioxane nanoparticles (BPS) with photocleavable *o*-nitrobenzyl bridged segments were designed and prepared. The silanol groups have the negative surface charges of BPS in an aqueous environment. The negatively charged BPS can attract positively charged drugs via electrostatic interactions. Upon irradiation, the photocleavage reaction of the organic bridged segments results in protonated amine groups which reverse the surface charge of BPS from the negative property to the positive property. At that time, the electrostatic equilibrium is unstable, so the electrostatic repulsion force results in the release of the target molecules from the surface of nanocarriers. Once the light is off, a new electrostatic equilibrium is established. Thus, a light-triggered, stepwise, and precisely controlled molecule release system can be fabricated. Doxorubicin (DOX), a positively charged drug, was used as a target molecule model compound to be loaded onto the negatively charged BPS. By regulating the irradiation intensity, time and on/off manner, multiple profiles of drug release and precise control of release timing, location, and dosage are reported. The BPS nanocarrier is an excellent candidate for the precisely controlled release of charged target molecules including food additive, dyes, cosmetics, pesticides, functional ultraviolet absorbents, and drugs in certain circumstances. However, since the use of the light wavelength at 254 nm has inevitable limitation such as harm to humans and weak penetration though skin, near-infrared or infrared light should be further considered to trigger the precisely controlled molecule release system.

## 2. Materials and Methods

### 2.1. Materials

*o*-Nitrobenzyl alcohol (98%), *N*,*N*-diisopropylethylamine (DIPEA, 98%), triphosgene (98%), bis(trimethoxysilylpropyl)amine (98%) were obtained from Shanghai Macklin Biochemical Co., Ltd. (Shanghai, China). Doxorubicin hydrochloride (DOX‧HCl) was purchased from Wuhan Dongkang Source Technology Co., Ltd. (Wuhan, China). Triethylamine (Et_3_N), hexane, dichloromethane (DCM), petroleum ether (PE), tetrahydrofuran (THF), ethyl acetate (EtOAc), isopropyl alcohol (*i*-PrOH), and methanol (MeOH) were sources from Sinopharm Chemical Reagent Co., Ltd. (Beijing, China). All organic solvents were distilled over a suitable drying reagent before use. All other reagents were analytical reagent grade and were purchased from Tianjin fuyu fine chemical Co., Ltd. (Tianjin, China).

### 2.2. Methods

#### 2.2.1. Synthesis of *o*-Nitrobenzyl Chloroformate

*o*-Nitrobenzyl alcohol (7.60 g, 49.6 mmol) and DIPEA (10.0 mL, 57.4 mmol) were dissolved in dry DCM (50.0 mL). Triphosgene (5.68 g, 19.2 mmol) was dissolved in dry DCM (20.0 mL) and added dropwise to the above mixture within 30 min at 0 °C. The reaction mixture was stirred at room temperature for 12 h. The resulting brownish solution was washed with water and extracted three times with DCM. The combined organic extracts were dried over anhydrous MgSO_4_. The organic solvent was removed under reduced pressure to give a brown crude product. The crude product was purified by column chromatography using DCM/PE (*v*/*v*, 3/1) as the eluent to yield *o*-nitrobenzyl chloroformate as a yellow solid (6.95 g, 32.3 mmol, 65.2% yield). Its chemical structure was confirmed by ^1^H NMR and ^13^C NMR spectra (Supplementary Materials, Figures S1 and S2).

#### 2.2.2. *o*-Nitrobenzyl Bis-trimethoxysilylpropyl Carbamate (*o*–NB)

Bis(trimethoxysilylpropyl)amine (6.82 g, 20.0 mmol) and Et_3_N (3.50 mL, 25.0 mmol) were dissolved in 40.0 mL of dry THF. The mixture was cooled at 0 °C, then was added *o*-nitrobenzyl chloroformate (4.30 g, 20.0 mmol) dropwise and dissolved in THF over 40 min. The reaction mixture was stirred at room temperature for 12 h. The mixture was filtrated to remove the Et_3_NH^+^Cl^–^ salts. Column chromatography (hexane/EtOAc, *v*/*v*, 7/4) was used to purify the condensed crude product to gain *o*-nitrobenzyl bis-trimethoxysilylpropyl carbamate (*o*–NB) as a yellow oil (7.12 g, 13.7 mmol, 68.5% yield). NMR (^1^H NMR, ^13^C NMR, and ^29^Si NMR) and MS spectra confirmed the chemical structure of *o*–NB (Supplementary Materials, Figures S3–S6).

#### 2.2.3. Bridged Polysilsesquioxane Nanoparticles (BPS)

A catalyst solution of 0.1 M NaOH (1.0 mL), isopropyl alcohol (1.0 mL) and H_2_O (18.0 mL) was added to a 50.0 mL round–bottom flask. The solution was kept at 80 °C, followed by the addition of *o*–NB (0.1 mL) under vigorous stirring. The mixture solution was stirred vigorously for 2 h. The sample was then washed three times with ethanol, deionized water, and ethanol, and dried under vacuum for a few hours.

#### 2.2.4. Photoreaction of the *o*–NB

An 80 μg/mL solution of *o*–NB in THF was placed in a volumetric flask at 10 cm using a bandpass filter (λ = 254 nm, 200 mW/cm^2^). 6.0 mL of solution was taken out at various time for UV–vis spectrum irradiation.

A solution of *o*–NB in CDCl_3_ (10.0 mg/mL) was irradiated at various times for the ^1^H NMR spectra. The parameters of the laser source, as well as the distance between laser and samples, were kept the same for all irradiation processes in this work.

#### 2.2.5. Light-Triggered Charge Reversal of BPS

A suspension of BPS (1 mg/mL in pH 7.4 PBS buffer solution) was placed in a volumetric flask and then irradiated for various durations. 6.0 mL of solution was taken out at various times for UV–vis and zeta potential characterization.

#### 2.2.6. Drug Loading and Light-Triggered Drug Release In Vitro

DOX (5.0 mg) was dissolved in 5.0 mL deionized water, followed by the addition of 10.0 mg BPS in 25.0 mL PBS buffer solution (pH 7.4) under stirring at room temperature for 24 h. The mixture was centrifuged at 8000 rpm for 20 min and washed with deionized water to remove the unloaded DOX until the supernatant became colorless. The obtained DOX–loaded BPS was named DOX@BPS. The UV–vis spectrum at 480 nm was used to determine the amount of the drug DOX loaded to the BPS based on a standard calibration curve obtained from free DOX in deionized water. The drug loading content (DLC) and drug loading efficiency (DLE) were calculated based on the following equations:

(1) $$\mathrm{DLC}\;(\%)=\frac{\mathrm{Weight}\;\mathrm{of}\;\mathrm{drug}\;\mathrm{in}\;\mathrm{nanoparticles}}{\mathrm{Weight}\;\mathrm{of}\;\mathrm{drug}\;\mathrm{loaded}\;\mathrm{nanoparticles}}\;\times \;100\%$$

(2) $$\mathrm{DLE}\;(\%)=\frac{\mathrm{Weight}\;\mathrm{of}\;\mathrm{drug}\;\mathrm{in}\;\mathrm{nanoparticles}}{\mathrm{Weight}\;\mathrm{of}\;\mathrm{drug}\;\mathrm{feeding}}\;\times \;100\%$$

For the light-triggered drug release behavior study in vitro, the dried DOX@BPS were dispersed into a PBS buffer solution (pH 7.4) at a concentration of 0.1 mg/mL in a 100.0 mL round–bottom flask. The suspension liquid was irradiated by different laser power densities of 60, 160, and 200 mW/cm^2^. At every time interval, 10.0 mL of the suspension liquid was taken out and centrifuged (8000 rpm) for 20 min; and the amount of DOX released from DOX@BPS were determined by the UV–vis method.

For the light-on or -off DOX release experiments, the sample suspensions were exposed to a laser power density of 200 mW/cm^2^ for 1 h (light on) and were then shielded for 1 h (light off), respectively. The light on/off experiments were repeated six times in 12 h. The release of DOX at each light on and light off event was measured by centrifugation (8000 rpm) for 20 min and the resulting DOX content was determined by the UV–vis method.

All the values were measured three times.

The rate of the released drug (%) was calculated based on the following equation:

(3) $$\mathrm{Rate}\;\mathrm{of}\;\mathrm{the}\;\mathrm{released}\;\mathrm{drug}\;\left(\%\right)=\frac{\mathrm{Weight}\;\mathrm{of}\;\mathrm{drug}\;\mathrm{in}\;\mathrm{the}\;\mathrm{PBS}}{\;\mathrm{Initial}\;\mathrm{weight}\;\mathrm{of}\;\mathrm{drug}\;\mathrm{in}\;\mathrm{the}\;\mathrm{nanoparticles}}\;\times \;100\%$$

### 2.3. Characterization Techniques

Nuclear magnetic resonance (NMR) spectra were recorded on a Bruker Avance 400 MHz spectrometer by using CDCl_3_ as the solvent and tetramethylsilane (TMS) as the internal standard.

Fourier transform infrared (FTIR) spectra were recorded on a Bruker Tensor 27 spectrometer in the wavenumber range of 400–4000 cm^–1^. Samples were ground with KBr and pressed to the plates for measurement.

The particle size, size distribution, and zeta potential were measured by light scattering using Malvern Zetasizer Nano ZS90 system. The diameter of NPs was received from the average of three measurement results.

The morphologies of the nanoparticles were observed by scanning electron microscopy (SEM, Hitachi SU8010) after drying and spraying Pt, and by transmission electron microscopy (TEM, Hitachi 2100) at 100.0 kV.

Thermal gravimetry analysis (TGA) was performed at a heating rate of 10 °C/min under a N_2_ atmosphere with a Thermo Gravimetric Analyzer (Netzsch STA 449).

Ultraviolet–visible (UV–vis) absorption spectra mesurements were performed on a Hitachi U–4100 UV–vis spectrometer.

## 3. Results and Discussion

### 3.1. Synthesis and Characteristic of Photoresponsive o–NB

**Scheme 1:**
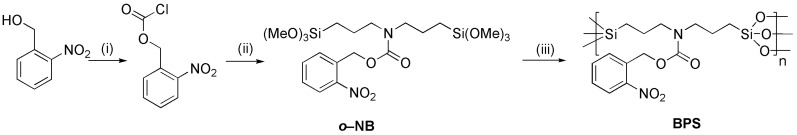
Synthesis of photoresponsive *o*–NB and BPS. Reaction conditions: (i) triphosgene, DIPEA, DCM, 0 °C; (ii) bis(trimethoxysilylpropyl)amine, Et_3_N, THF, 0 °C; (iii) NaOH, *i*-PrOH, 80 °C, 1500 rpm.

As illustrated in Scheme 1, *o*-nitrobenzyl alcohol reacted with triphosgene at 0 °C via a substitution reaction to obtain *o*-nitrobenzyl chloroformate. Then the intermediate product and bis(trimethoxysilylpropyl)amine underwent a substitution reaction in an ice-water bath to produce a bridged siloxane with photoresponsive *o*-nitrobenzyl groups (*o*–NB). The chemical structures of all compounds were confirmed via nuclear magnetic resonance (NMR) and mass spectra (MS) spectroscopies (Figures S1–S6).

**Figure 1:**
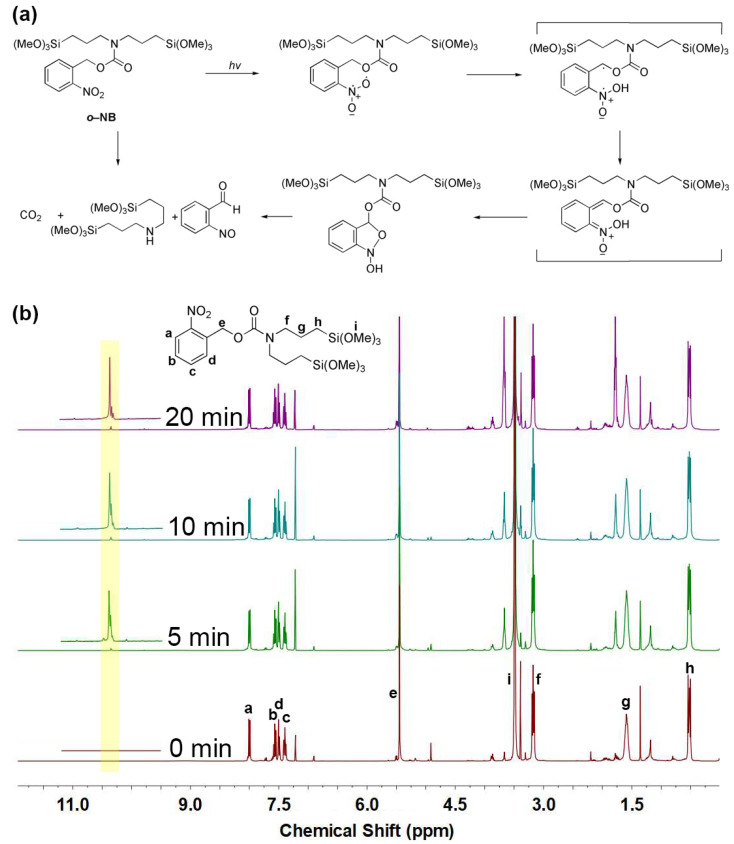
(**a**) The photolysis mechanism of *o*–NB upon irradiation at 254 nm. (**b**) Comparison of the ^1^H NMR spectra of *o*–NB after irradiation 0, 5, 10, and 20 min, depicting its photoresponsive behavior with the disappearance of the benzyl peak at 5.4 ppm (Ph–*CH*_2_–).

The pendent *o*-nitrobenzyl ester group of the monomer underwent photocleavage into a corresponding *o*-nitrosobenzaldehyde upon light irradiation at 254 nm, simultaneously releasing CO_2_ (Figure 1a). ^1^H NMR spectra was used to prove the photocleavage of *o*–NB. Figure 1b shows the ^1^H NMR spectra of the *o*–NB in CDCl_3_ before and after light irradiation. The integral of peak labeled “f” at 3.21 ppm attributed to H(N–*CH_2_*–) was set as 4.0. The integral of peak labeled “e” at 5.4 ppm belongs to protons on the benzyl group gradually decreasing from 2.00 to 1.75 with the increasing irradiation time, indicating the consumption of benzyl groups after the photocleavage of the *o*-nitrobenzyl ester groups. Meanwhile, the appearance of a new peak at 10.3 ppm suggested the formation of photolysis product aldehyde groups. No observed visible changes occurred in the protons attributed to the benzene ring (a~d) and aliphatic alkyl (f~i).

**Figure 2:**
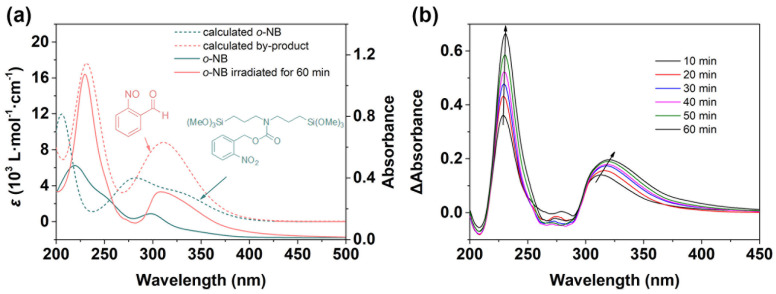
(**a**) The measured (solid line) and theory calculated (dotted line) UV–vis spectra of *o*–NB in THF solution under light irradiation at 254 nm. (**b**) UV–vis absorption spectra of light irradiated *o*–NB for different times.

The photoresponsive behavior of *o*–NB in the tetrahydrofuran solution (80 μg/mL) was monitored by UV–vis spectroscopy (Figure 2). UV–vis spectrum of the unirradiated *o*–NB in THF (the solid green line in Figure 2a) showed the characteristic absorption of the π→π* electron transition at 220 nm and the n→π* electron transition at 294 nm, belonging to benzyl groups, nitryl, and ester groups, respectively. After irradiation for 60 min (solid red line in Figure 2a), the absorption at 230 nm and 307 nm corresponded to the π→π* of benzyl groups and the n→π* of nitroso and aldehyde groups, respectively, due to the consumption of *o*–NB and the generation of the photolysis by-product *o*-nitrosobenzaldehyde. The red shiftsindicated the photoresponsive character of *o*–NB, which was further confirmed by theory calculated UV−vis absorption spectra of *o*–NB and *o*-nitrosobenzaldehyde (dotted line in Figure 2a). The characteristic absorption peaks of *o*–NB at about 220 and 300 nm gradually shifted and increased over the irradiation times. The difference between the two UV−vis absorption spectra of irradiated *o*–NB and unirradiated *o*–NB shows the evolution of the photocleavage reaction in detail (Figure 2b).

### 3.2. Synthesis and Characteristic of BPS

**Figure 3:**
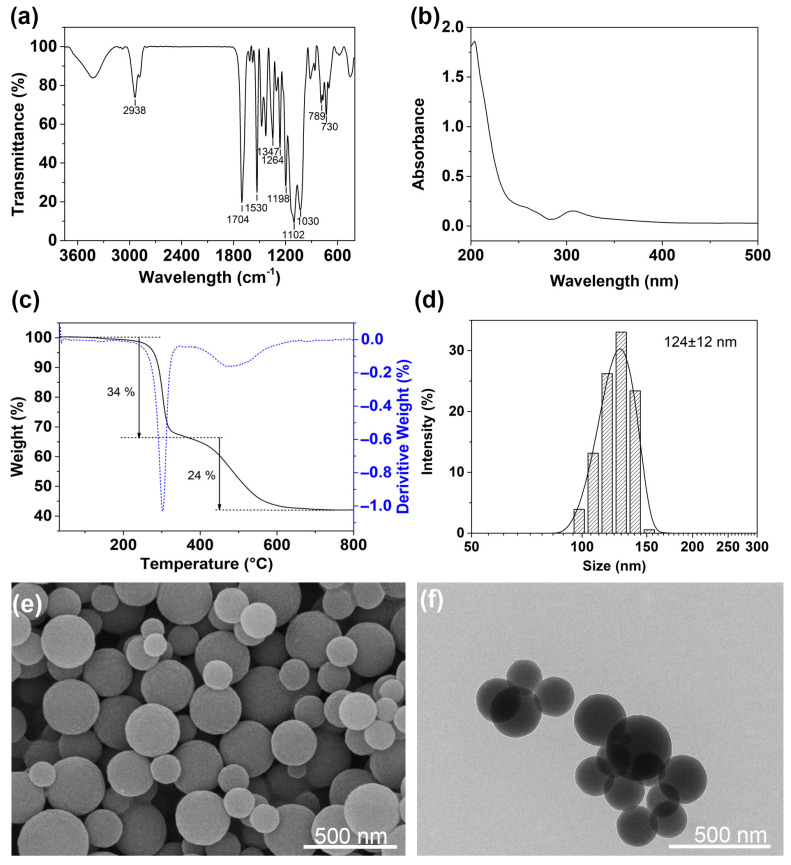
FTIR (**a**), UV–vis (**b**), TGA (**c**), and DLS (**d**) spectra of BPS. SEM (**e**) and TEM (**f**) micrographs of BPS.

*o*-Nitrobenzyl bis-trimethoxysilylpropyl carbamate bridged polysisesquioxane nanoparticles were synthesized via a sol-gel process using sodium hydroxide as a basic catalyst and *i*-PrOH as a cosolvent. The hydrolysis-condensation reaction of the *o*–NB monomer was conducted under vigorous stirring at 80 °C for 2 h. The nanoparticles were collected by centrifugation, washed with deionized water and ethanol, and dried under vacuum 4 h, giving a white powder. The photoresponsive BPS was carefully characterized by FTIR, UV–vis, and TGA measurements (Figure 3). The FTIR spectrum of BPS (Figure 3a) showed the Si–O stretching vibration mode at 1102 cm^−1^ and 1030 cm^−1^. The Si–C stretching vibration mode at 1198 cm^−1^ indicated that the organic bridged segments covalently incorporated into the polysisesquioxanes. The peak at 2938 cm^−1^ was attributed to the C–H stretching vibration. The presence of –NO_2_ groups was proven by the typical peaks at 1540 cm^−1^ of antisymmetric stretching vibration and at 1365 cm^−1^ symmetric stretching vibration, respectively. The stretching vibration of C=O at 1705 cm^−1^ and C–O at 1264 cm^−1^ revealed the eater groups in the organic bridged segments. In addition, the C–H bending vibration of the phenyl groups appeared at 789 cm^−1^ and 730 cm^−1^. The UV–vis absorption spectra (Figure 3b) of BPS dispersed in PBS buffer solution (pH 7.4) showed the characteristic adsorption of the π→π* electron transition at 203 nm attributed to the benzene ring and that of the n→π* electron transition at 306 nm attributed to the unsaturated –NO_2_ and C=O groups, demonstrating the possibility of the photoresponsiveness of BPS with *o*-nitrobenzyl ester organic groups. The difference between the UV–vis absorption spectra of the BPS and the *o*–NB may come from the effect of Si–O–Si network on *o*-nitrobenzyl ester groups. The TGA curves were analyzed to quantify the organic content of BPS (Figure 3c). The total weight loss suggested 58.0 wt% of BPS. The first stage of weight loss before 370 °C was caused by the thermal decomposition of the pendant *o*-nitrobenzyl ester groups. The second stage of weight loss from 370 °C to 700 °C arose from the thermal decomposition of aliphatic bridged chains. The two-stage profiles of weight loss were also clearly shown by the DTG curves (blue dotted line in Figure 3c). The dynamic light scattering (DLS) results showed that the average size of the BPS was 124 ± 12 nm (Figure 3d). The spherical shape of the prepared BPS nanoparticles were confirmed by SEM and TEM (Figure 3e,f).

### 3.3. Light-Triggered Charge Reversal of BPS

**Figure 4:**
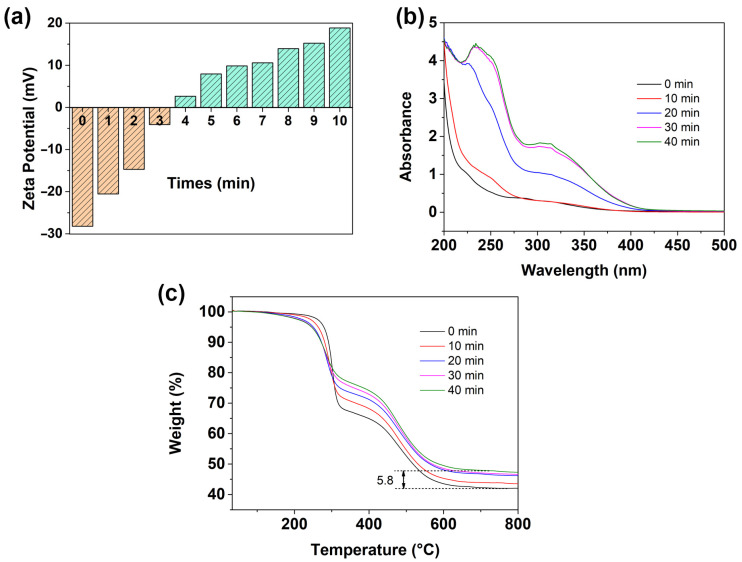
Zeta potential (**a**), UV–vis absorption spectra (**b**), and TGA curves (**c**) of BPS upon light irradiation for various times.

The photoresponsive behavior of BPS was investigated using the Zeta potential, DLS, TGA, and UV–vis methods. Zeta potential measurements in PBS solution (pH 7.4) on BPS revealed a negative charge property of −28.20 mV (Figure 4a). In a typical experiment, when an aqueous suspension of BPS was irradiated at 254 nm (200 mW/cm^2^) for 10 min, the surface charge property reversed to +18.9 mV (Figure 4a), indicating the light-triggered surface charge reversal feature of BPS. In fact, after irradiating for 4 min, the surface charge property of the nanoparticles had already reversed to a positive state (+2.64 mV), suggesting the fast responsiveness of BPS to light. The light-triggered surface charge reversal property resulted from the photocleavage of the *o*-nitrobenzyl groups. Before irradiation, the existence of silanol groups on the surface of the BPS resulted in a negatively charged surface. Upon irradiation, secondary amine groups were exposed in the bridged segments after the photoreaction of the *o*-nitrobenzyl groups. The protonation of the produced amine groups reversed the surface charge of the nanoparticles from a negative to a positive state. Figure 4b shows the TGA measurements of the BPS over different irradiation times. The char yield of the BPS was 42.1 wt% under N_2_ atmosphere. However, the char yield of the BPS irradiated for 40 min increased to 47.9 wt%. The 5.8 wt% difference in char yield was attributed to the photocleavage of the *o*-nitrobenzyl groups of the BPS. The supernatant of BPS over different irradiation times was monitored by the UV–vis method (Figure 4c). The increasing absorption peaks at 234 nm and 306 nm corresponded to the π→π* and n→π* electron transitions of the photolysis by-product *o*-nitrosobenzaldehyde. In addition, the supernatant of BPS turned gradually from colorless to slightly yellow due to the by–product *o*-nitrosobenzaldehyde being produced upon light irradiation.

### 3.4. Drug Loading and Light-Triggered Drug Release In Vitro

**Figure 5:**
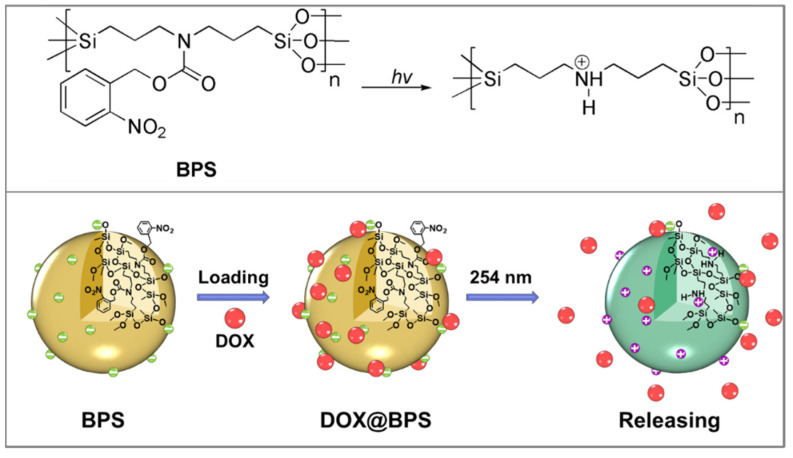
The photocleavage reaction of BPS (**top**). Schematic illustration of DOX@BPS target molecules release (**down**).

Light-triggered charge reversal BPS is a promising candidate for the precise control of molecule release. DOX, a clinical anticancer drug, was used to evaluate the light-triggered molecule release system. As shown in Figure 5, the positively charged DOX was loaded onto the negatively charged BPS (DOX@BPS) by electrostatic interaction in PBS buffer solution (pH 7.4). Upon irradiation at 254 nm, the charge reversal of BPS from a positive to a negative state created electrostatic repulsion between DOX and BPS, resulting in the release of the DOX.

**Figure 6:**
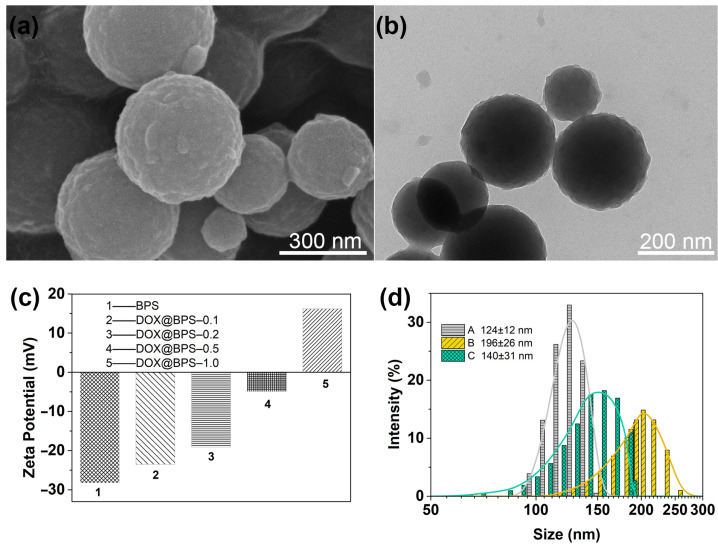
SEM (**a**) and TEM (**b**) micrographs of DOX@BPS; (**c**) zeta potential of DOX@BPS prepared with different feed ratios; (**d**) size of BPS (A), DOX@BPS (B) and DOX@BPS after drug release in PBS buffer solution (pH 7.4) upon light irradiation (C).

The SEM and TEM micrographs showed that the surface of DOX@BPS became rough, indicating the target molecules of DOX had loaded on the BPS (Figure 6a,b).The zeta potential of DOX@BPS was studied in the preparation process (Figure 6c). When the feed ratio of DOX to BPS was 0.5, the zeta potential of the obtained DOX@BPS maintained a negatively charged state of −4.9 mV. With the increasing feed ratio of DOX, the surface charge of DOX@BPS reversed to positively charge at +16.2 mV. Due to negatively charged nanocarriers having more advantage in a drug release system, the feed ratio of 0.5 of DOX to BPS was used to prepared the DOX@BPS. The loading content and entrapment efficiency of DOX@BPS at a 0.5 feed ratio was 12.8% and 67.3%, respectively. Furthermore, the average size of DOX@BPS increased to 196 ± 26 nm due to the DLS (Figure 6d). The results of the zeta potential, morphologies, and increased average size illustrated that target molecules DOX had been successfully loaded on the BPS.

**Figure 7:**
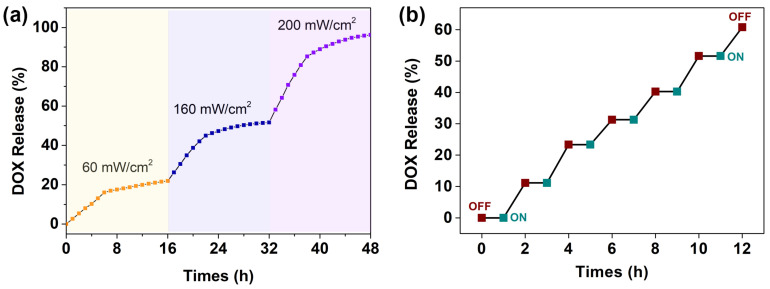
(**a**) DOX release profiles of DOX@BPS at different laser intensities in PBS buffer solution (pH 7.4) at 37 °C. (**b**) Progress of release of DOX under light irradiation and dark conditions; “ON” indicates the beginning of light irradiation and “OFF” indicates the ending of light irradiation.

The DOX release profile of DOX@BPS from irradiation times of 0 to 16 h at different power densities of 60 mW/cm^2^, 160 mW/cm^2^ and 200 mW/cm^2^ were evaluated (Figure S8). DOX release of DOX@BPS under dark conditions after 16 h was only 3.5%, indicating DOX@BPS had good stability without irradiation. When the power density of the laser was 60 mW/cm^2^, 160 mW/cm^2^, and 200 mW/cm^2^, the amounts of DOX released under irradiation 16 h were 21.9%, 48.9% and 82.9%, respectively. To continuously enhance the cumulative release of DOX@BPS, the DOX release behaviors under changed light power densities (60, 160 and 200 mW/cm^2^) over irradiation times from 0 to 48 h in PBS buffer solution (pH 7.4) at 37 °C were further studied (Figure 7a). UV–vis absorption spectroscopy recorded the absorbance of the supernatant of DOX@BPS upon light irradiation for different durations. The released DOX amounts increased remarkably with increased light irradiation density, suggesting that the target molecule release rate and the cumulative release amount could be regulated in a light-controlled manner by changing the light irradiation intensity and irradiation time. For example, the released DOX in PBS buffer solution (pH 7.4) at a 60 mW/cm^2^ power density was only 21.8% of loaded DOX for light irradiation at 16 h. By contrast, at a 120 mW/cm^2^ power density, the amount of released DOX was 51.7% after 16 h. Enhancing the irradiation power density to 200 mW/cm^2^ induced a 96.2% drug release of the DOX@BPS. Figure 7a clearly shows that a high photoreaction conversion efficiency under a high intensity of laser light resulted in a faster DOX release rate. As a note, a two-stage profile with an initially higher rate of release that thereafter leveled off was clearly shown at the same irradiation power density. No burst release was observed in any irradiation period, because the driving force of the target molecule release was electrostatic interaction instead of the concentration diffusion mechanism. The average size of the DOX@BPS decreased to 140 ± 31 nm after DOX release in the PBS buffer solution (pH 7.4) (Figure 6d).

To confirm the capability of a precisely controlled release, a turning on/turning off mode of the target molecule release behavior of DOX@BPS was performed in PBS buffer solution at 37 °C upon irradiation at a 200 mW/cm^2^ power intensity. The release process was monitored by UV–vis absorption spectroscopy. During the first laser on and laser off unit operation (1 h), 11.1% of DOX was released from DOX@BPS. DOX release from DOX@BPS only proceeded under irradiation. No DOX release was detected under dark conditions. Light-triggered target molecule release behavior was observed when the same irradiation time was repeated every one hour, and sustained release of DOX up to 60.8% was detected after irradiating DOX@BPS for 6 h. The typical “stepped” profile indicated that the target molecules DOX can be released in a triggered, stepwise, and precisely controlled manner.

## 4. Conclusions

In conclusion, a light-triggered charge reversal bridged polysilsesquioxane nanoparticle was successfully designed and prepared. Upon light irradiation, the negatively charged (–28.20 mV) polysilsesquioxane nanoparticles can reverse their surface charge to positive state (+18.9 mV) due to the photocleavage of the organic bridged segments. This nanocarrier was considered an ideal platform for precise control of target molecule release. Doxorubicin, a positively charged model molecule, could be loaded on the surface via electrostatic interaction. The charge reversal property of the nanoparticles could release the loaded target molecules. The release kinetics of the target molecules depended on the intensities, times, and modes of light irradiation. A nearly complete DOX release (96%) was achieved by regulating light irradiation intensity and time. The typical stepped profile of DOX release under an on/off light irradiation mode provides a light-triggered, stepwise, and precisely controlled approach to target molecule release. This precisely controlled release system has potential applications in the fields of food additives, pesticides, dyes, cosmetics, functional ultraviolet absorbents and drugs in certain circumstances. Further research should attempt to use NIR or IR light to trigger the precisely controlled molecule release system to overcome the limitation of UV light at 254 nm for biomedical applications.

## Data Availability

The data presented in this study are available on request from the corresponding author.

## Supplementary Materials

The following are available online at https://www.mdpi.com/article/10.3390/polym13152392/s1, Figure S1: ^1^H NMR spectrum of *o*-nitrobenzyl chloroformate, Figure S2: ^13^C NMR spectrum of *o*-nitrobenzyl chloroformate, Figure S3: ^1^H NMR spectrum of *o*-nitrobenzyl bis-trimethoxysilylpropyl carbamate, Figure S4: ^13^C NMR spectrum of *o*-nitrobenzyl bis-trimethoxysilylpropyl carbamate, Figure S5: ^29^Si NMR spectrum of *o*-nitrobenzyl bis-trimethoxysilylpropyl carbamate, Figure S6: MS spectrum of *o*-nitrobenzyl bis-trimethoxysilylpropyl carbamate, Figure S7: The calculated chemical structures of *o*–NB monomer (**a**) and photolysis by-product *o*-nitrosobenzaldehyde (**b**), Figure S8: DOX release profiles of DOX@BPS from 0 to 16 h at different laser intensities (0, 60,160, and 200 mW/cm^2^) in PBS buffer solution (pH 7.4) at 37 °C.
