# Supplementary material for: Precise Controlled Target Molecule Release through Light-Triggered Charge Reversal Bridged Polysilsesquioxane Nanoparticles
Source: Polymers (Basel). 2021 Jul 21;13(15):2392. doi: 10.3390/polym13152392 (PMC8346980; doi:10.3390/polym13152392)
Supplement: Supplementary file 1 [file polymers-13-02392-s001.zip › polymers-1293326-supplementary.pdf]

polymers

Supplementary Material: Precise Controlled Target Molecule Release through Light-Triggered Charge Reversal Bridged Polysilsesquioxane Nanoparticles

Xin Zhang, Mengmeng Zhang, Mingyue Wu, Linchuan Yang, Rui Liu, Rui Zhang, Tongtong Zhao, Ci Song, Gang Liu and Qingzeng Zhu *

Key Laboratory of Special Functional Aggregated Materials, Ministry of Education, School of Chemistry and Chemical Engineering, Shandong University. Jinan, 250100, China;

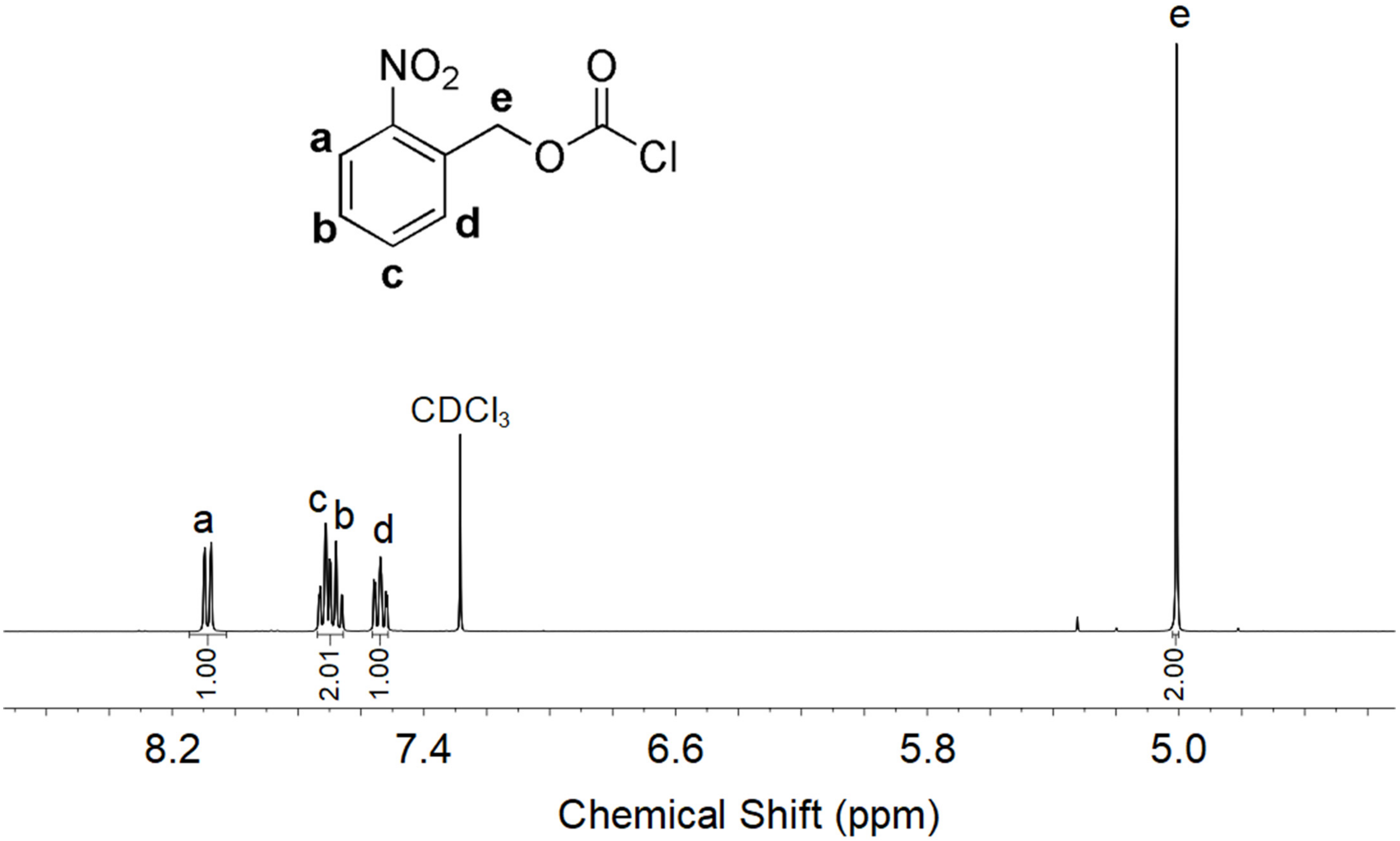

Figure S1. ^{1}H NMR spectrum of *o*-nitrobenzyl chloroformate.

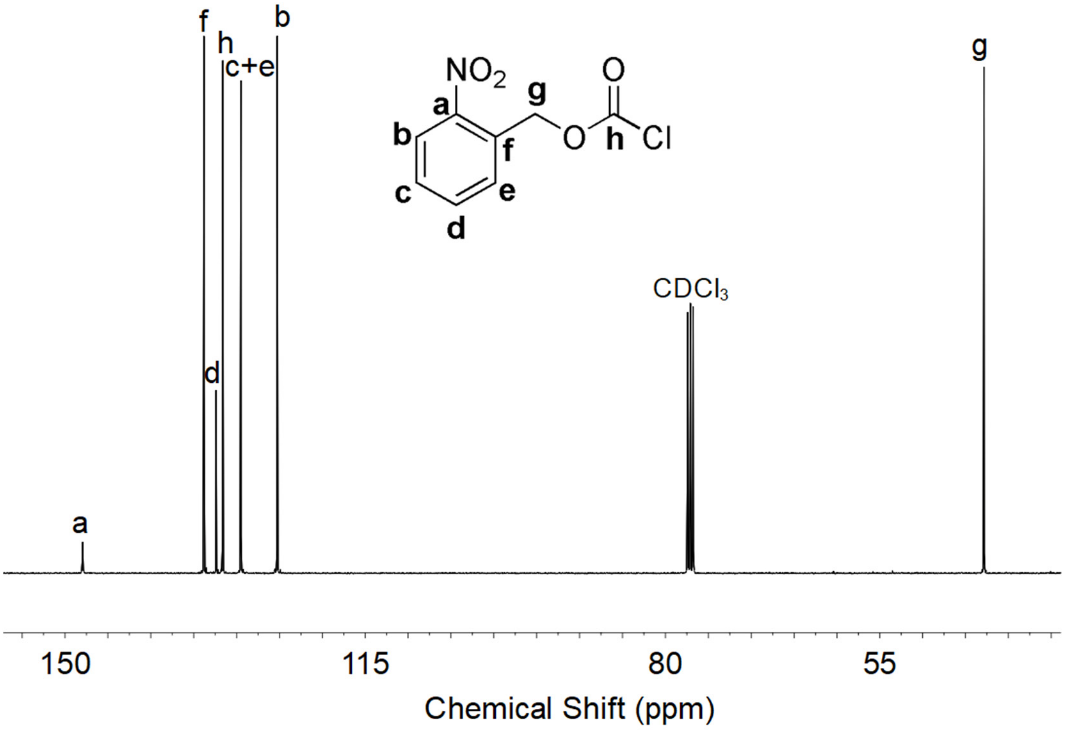

Figure S2. ^{13}C NMR spectrum of *o*-nitrobenzyl chloroformate.

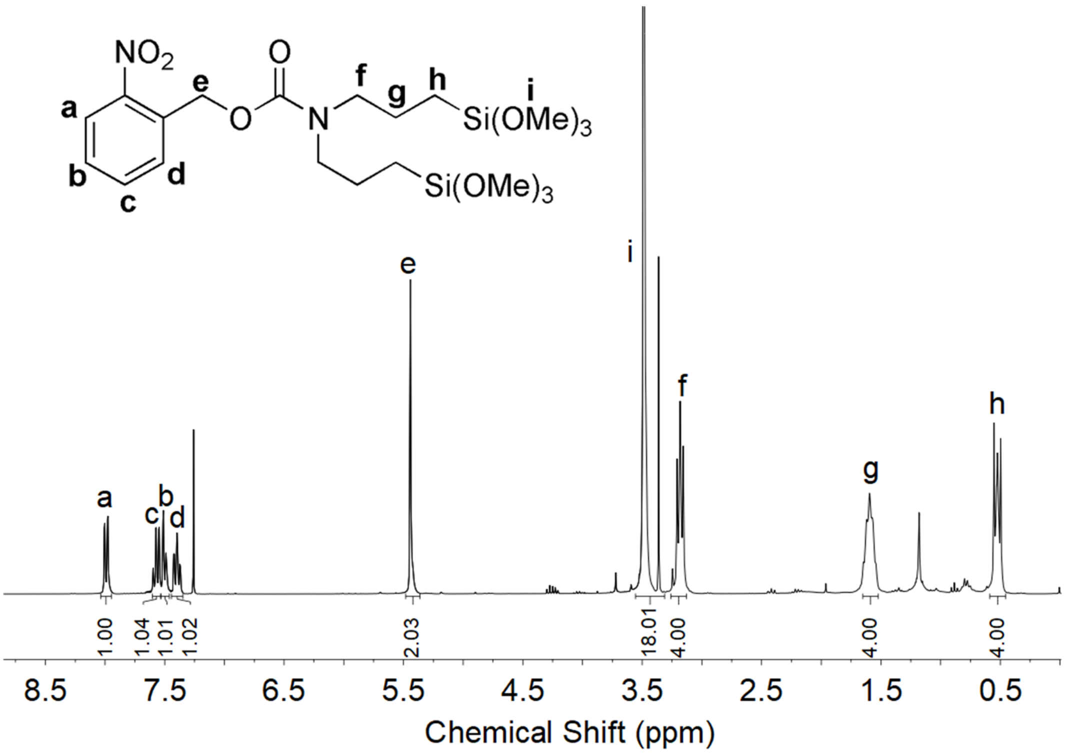

Figure S3. ^{1}H NMR spectrum of *o*-nitrobenzyl bis-trimethoxysilylpropyl carbamate.

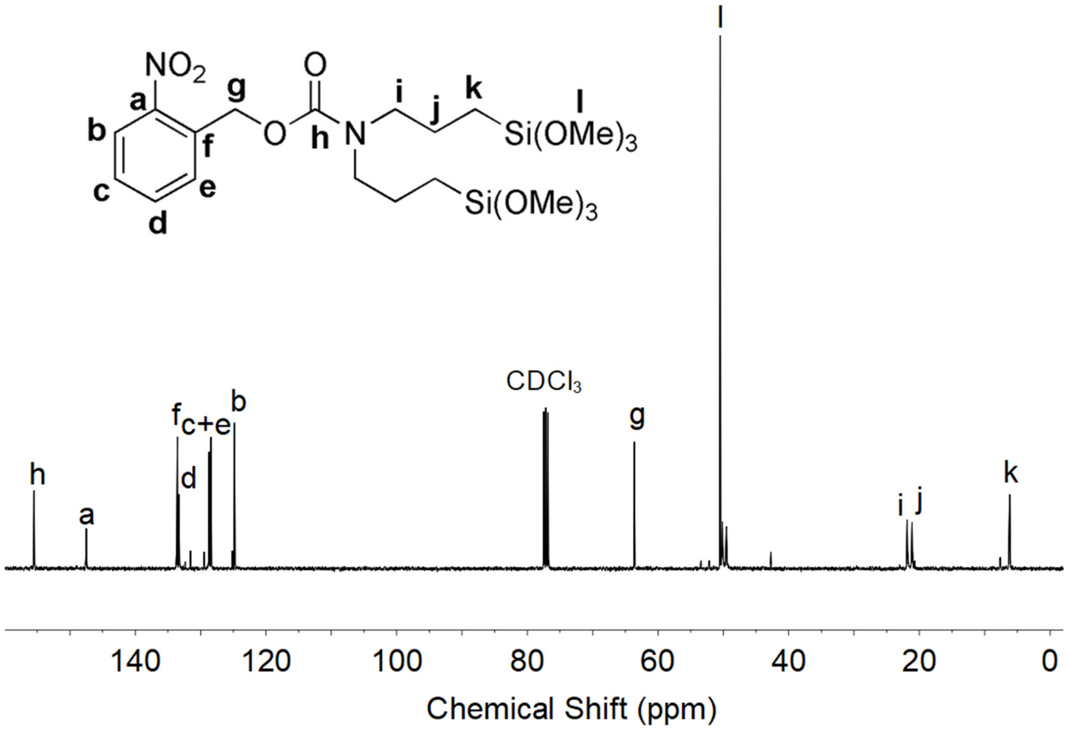

Figure S4. ^{13}C NMR spectrum of *o*-nitrobenzyl bis-trimethoxysilylpropyl carbamate.

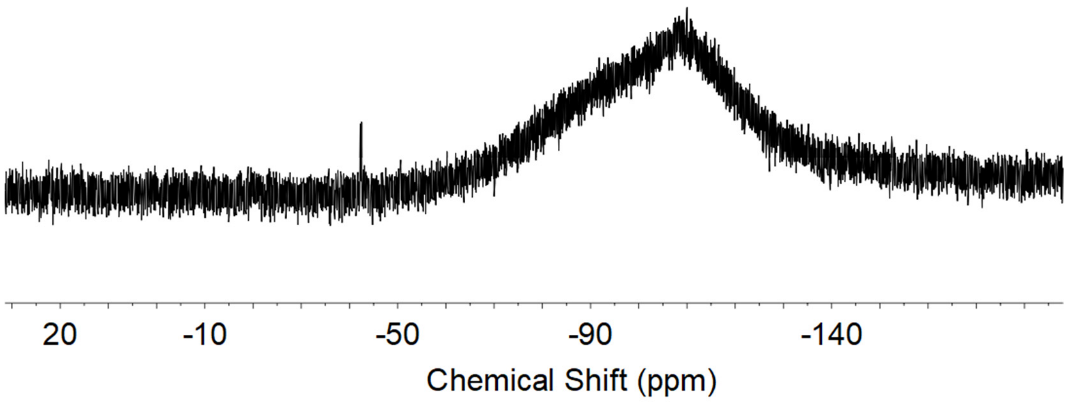

Figure S5. ^{29}Si NMR spectrum of *o*-nitrobenzyl bis-trimethoxysilylpropyl carbamate.

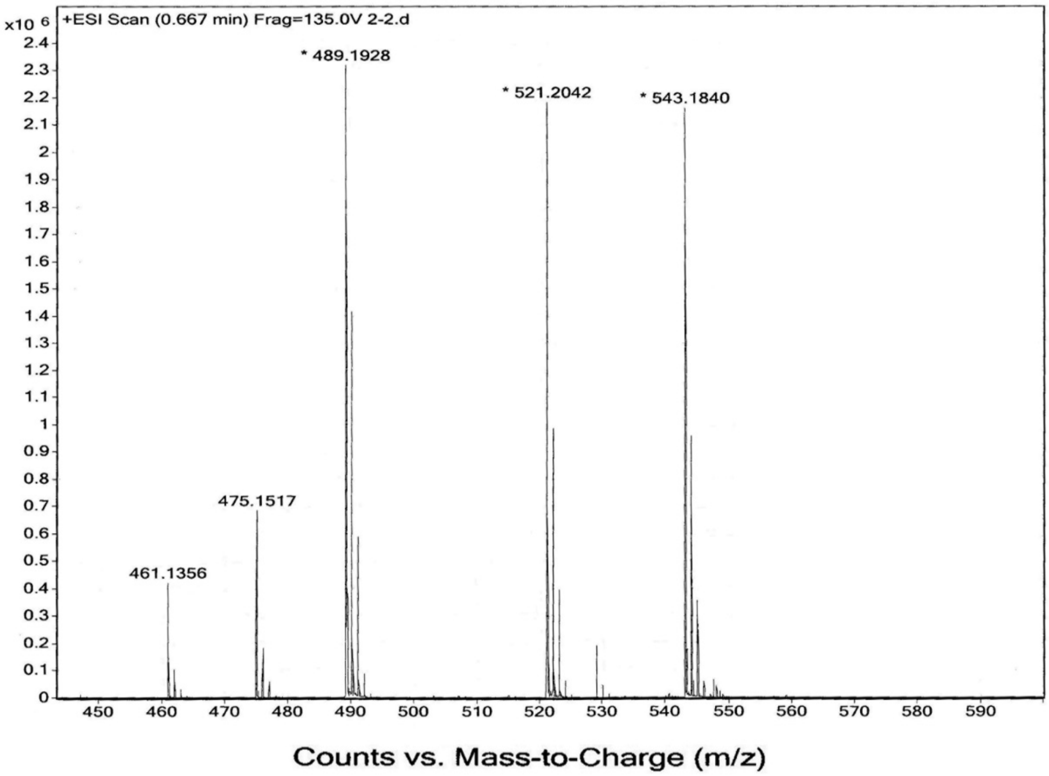

Figure S6. MS spectrum of *o*-nitrobenzyl bis-trimethoxysilylpropyl carbamate.

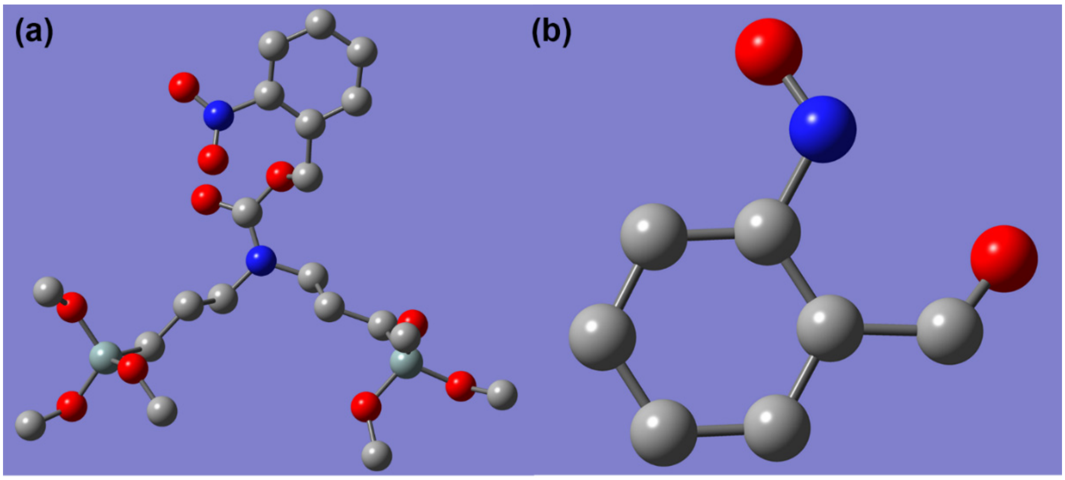

Figure S7. The theroy calculated chemical structures of *o*–NB monomer (a) and photolysis by-product *o*-nitrosobenzaldehyde (b).

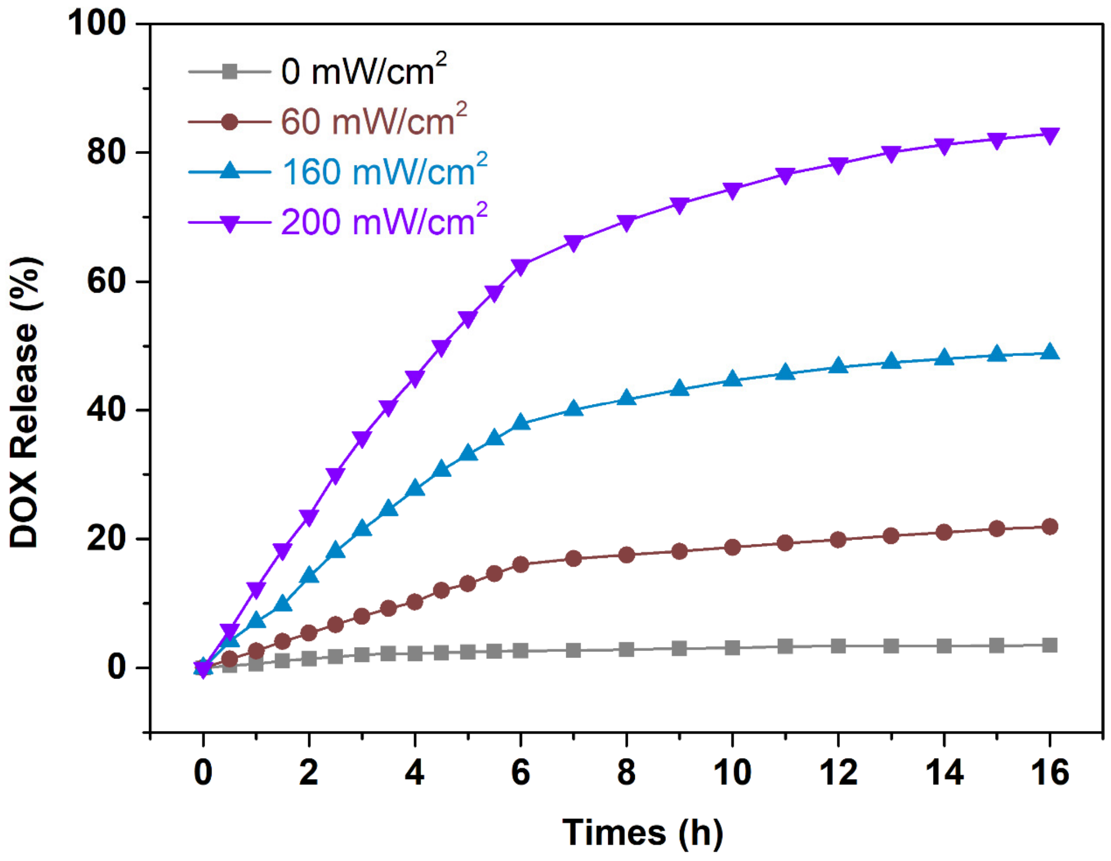

Figure S8. DOX release profiles of DOX@BPS from 0 to 16 h at different laser intensities (0, 60,160, and 200 mW/cm²) in PBS buffer solution (pH 7.4) at 37 °C.
